# Genome investigations show host adaptation and transmission of LA-MRSA CC398 from pigs into Danish healthcare institutions

**DOI:** 10.1038/s41598-019-55086-x

**Published:** 2019-12-09

**Authors:** Raphael Niklaus Sieber, Anders Rhod Larsen, Tinna Ravnholt Urth, Søren Iversen, Camilla Holten Møller, Robert Leo Skov, Jesper Larsen, Marc Stegger

**Affiliations:** 1 Statens Serum Institut, Department of Bacteria, Parasites & Fungi, Artillerivej 5, 2300 Copenhagen S, Denmark; 2 Statens Serum Institut, Department of Infectious Disease Epidemiology & Prevention, Artillerivej 5, 2300 Copenhagen S, Denmark

**Keywords:** Clinical microbiology, Epidemiology, Genome-wide association studies

## Abstract

Over the last decade, an increasing number of infections with livestock-associated methicillin-resistant *Staphylococcus aureus* of clonal complex 398 (LA-MRSA CC398) in persons without contact to livestock has been registered in Denmark. These infections have been suggested to be the result of repeated spillover of random isolates from livestock into the community. However, other studies also found emerging sub-lineages spreading among humans. Based on genome-wide SNPs and genome-wide association studies (GWAS), we assessed the population structure and genomic content of Danish LA-MRSA CC398 isolates from healthcare-associated infections from 2014 to 2016 (*n = *73) and compared these to isolates from pigs in Denmark from 2014 (*n* = 183). Phylogenetic analyses showed that most human isolates were closely related to and scattered among pig isolates showing that the majority of healthcare-associated infections are the result of repeated spillover from pig farms, even though cases of human-to-human transmission also were identified. GWAS revealed frequent loss of antimicrobial resistance genes and acquisition of human-specific virulence genes in the human isolates showing adaptation in response to changes in selective pressures in different host environments, which over time could lead to the emergence of LA-MRSA CC398 lineages more adapted to human colonization and transmission.

## Introduction

Human infections with livestock-associated methicillin-resistant *Staphylococcus aureus* of clonal complex 398 (LA-MRSA CC398) have increased dramatically over the last decade^1,2^ and accounted for 16% of all MRSA infections in Denmark in 2016^3^. Since 2012, the Danish MRSA guidelines have recommended screening of patients with livestock contact at hospital admission to limit the introduction of MRSA into healthcare institutions in Denmark^4^. However, an increasing number of people colonized or infected with LA-MRSA CC398 have no contact to livestock^1^. As opposed to livestock-onset (LO) MRSA infections, where a direct link to livestock is recorded, such cases of MRSA infection are categorized as either healthcare-onset (HO) if the positive culture was obtained ≥48 hours after admission to a healthcare facility, or community-onset (CO) if the positive culture was obtained from patients in the primary healthcare sector or within the first 48 hours of admission without having had contact to the healthcare institutions within the last 12 months. Furthermore, infections are categorized as healthcare-associated community-onset (HACO) if the patients have been admitted to a healthcare institution within the last 12 months before onset in the community. Because most of these non-LO cases have no risk factors for MRSA carriage (e.g., livestock contact), they may inadvertently introduce the bacteria into hospitals and nursing homes. This increases the risk of nosocomial transmission of LA-MRSA CC398 to other patients^5–7^, including immuno-compromised and elderly people where the bacteria can cause severe disease and occasionally death^2,8–10^.

In Denmark, the number of non-LO infections with LA-MRSA CC398 has been increasing in parallel with the number of LO infections^1^. This suggests a scenario where the general population is consistently exposed to a random spillover of bacteria from livestock, as described in earlier studies from Denmark^11^ and other European countries^12–14^. However, recent studies also report the emergence of sub-lineages spreading independently of the livestock reservoir^15,16^. Such lineages could be better adapted to the human host, which increases the risk of spread into healthcare institutions via non-LO cases with the present guidelines. Phylogenetic analysis based on whole-genome sequencing (WGS) is highly sensitive with regard to identifying emerging sub-lineages^17,18^ and has recently enabled the identification of three predominant lineages of LA-MRSA CC398 (termed L1, L2 and L3) in the Danish pig production system^11^. Furthermore, WGS can be used to detect genetic differences between isolates present in different host environments, such as the gain or loss of host-specific genes^17–22^.

The aims of this study were to: (i) determine whether the introduction of LA-MRSA CC398 into healthcare institutions is due to repeated spillover of random isolates from livestock or to circulation of sub-lineages with an increased capacity for human colonization and transmission; and (ii) investigate relevant bacterial genomes for signatures of adaptation to the human host.

## Results

### Temporal trends of human LA-MRSA CC398 infections in Denmark, 2007–2016

**Figure 1:**
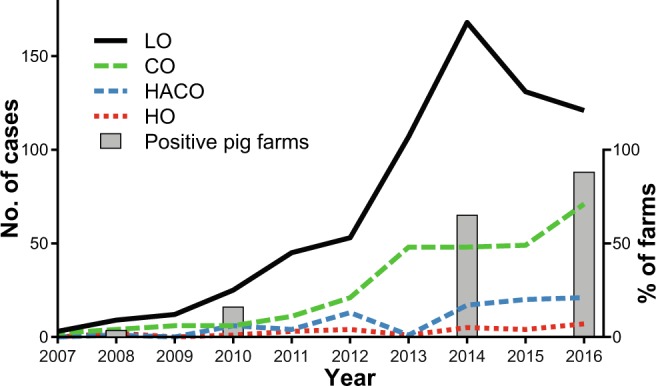
Trends in human CC398 LA-MRSA infections of different onset compared to its prevalence in livestock. The numbers of infections from 2007 to 2016 with hospital (HO, red line, and HACO, blue line), community (CO, green line) and livestock (LO, black line) are shown together with the prevalence on Danish pig farms (bars).

The number of LA-MRSA CC398 infections in Denmark increased from five in 2007 to 220 in 2016. Whereas the majority (64.2% [674/1050]) of cases from this period could be categorized as LO, 35.8% (376/1050) of the cases had no apparent contact to livestock. The latter were further differentiated into HO (2.6% [27/1050 cases]), HACO (7.9%, 83/1050 cases) and CO (the remaining 25.3% [266/1050] of cases). The annual numbers of CO cases increased in parallel with the prevalence of LA-MRSA CC398 in pig farms (Fig. 1), while the annual numbers of HO and HACO cases did not exceed seven and 21, respectively.

### Healthcare-associated LA-MRSA CC398 infections in Denmark, 2014–2016

In the 3-year period from January 2014 through December 2016, 73 infections were categorized as either HO (*n = *15) or HACO (*n = *58). The isolates were obtained from skin and soft tissue (*n = *49), nose and mouth, including tonsils and sinuses (*n = *8), respiratory sites, including tracheal aspirates, sputum and induced sputum (*n = *6), blood and cerebrospinal fluid (*n* = 4), ear or eye (*n* = 1), and other clinical sites, including indwelling devices and unspecified origin (*n = *5). The median age of the patients was 66 years (range, 0–97 years) and the female-to-male ratio was 0.87. The geographic distribution of human LA-MRSA CC398 isolates from healthcare-associated infections did not differ significantly from the distribution of pig isolates (Table 1).

**Table 1:** Distribution of LA-MRSA CC398 isolates from humans and pigs among the five Danish healthcare regions.

Region	No. (%) of isolates		*p* value
	Human	Pig	
The Capital Region of Denmark	5 (7)	n.d.	n.d.
Central Denmark Region	18 (25)	48 (26)	0.875
The North Denmark Region	24 (33)	47 (26)	0.28
The Region of Southern Denmark	22 (30)	70 (38)	0.25
Region Zealand	4 (5)	18 (10)	0.329
Total	73 (100)	183 (100)	

The numbers and percentages of isolates from healthcare-associated infection from 2014 through 2016 (*n* = 73) and from pigs from 2014 (*n* = 183) among the five Danish healthcare regions are displayed. The absence of any significance indicates that there is no difference in the geographical distribution between isolates from humans and pigs. Abbreviations: n.d., Not determined.

### Phylogenetic analysis of LA-MRSA CC398 isolates from pigs and people with healthcare-associated infections

**Figure 2:**
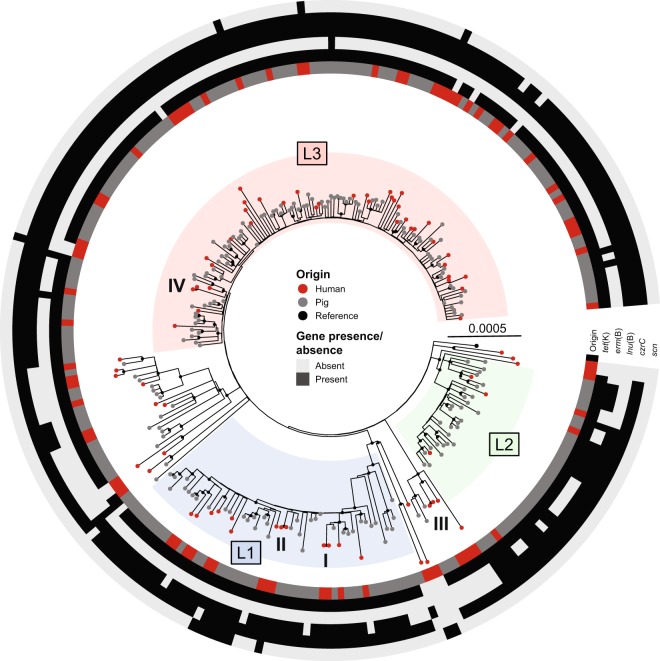
Phylogenetic tree of the 256 isolates from humans and pigs. Rooted maximum-likelihood tree of the derived CC398 livestock clade containing 73 isolates from people with healthcare-associated infections (red) and 183 isolates from Danish pig farms (grey). The four pairs of closely related human isolates are indicated by roman numbers and the three major lineages (L1–L3) within the Danish pig production are highlighted. The tree is based on 3,245 variable sites after filtering for recombination tracts (823 sites). Bootstrap values above 90% are illustrated by filled black circles at the nodes. The scalebar indicates substitutions per site.

A total of 73 isolates from healthcare associated human infections were compared to the 183 isolates from pig farms in a phylogenetic analysis. The maximum-likelihood phylogeny was established based on 3,245 variable sites after removal of 873 sites falling into recombinant regions. The phylogenetic analysis showed that human isolates were scattered among pig isolates, and only a few clusters of two to three human isolates were observed. The majority (78% [57/73] of isolates from patients with healthcare-associated infections was most closely related to a pig isolate with a median distance of 36 SNPs (range 3–58 SNPs). Of the remaining 16 human isolates, eight formed four pairs with a SNP distance of ≤2 while the last eight were most closely related to other human isolates but with a median distance of 16 SNPs (range 9–73 SNPs) between them (Fig. 2). All four pairs of isolates with ≤2 SNPs had identical regional origin, and in all four cases potential epidemiological links could be determined (Table 2). In three pairs, both patients had stayed in the same healthcare unit at the same time (pairs I-III), while both patients in the last pair shared the same home care unit (pair IV). Of note, two isolates from two distinct pairs (II and IV) contained the human immune evasion cluster (IEC) carried on the ΦSa3 prophage (Table 2). Whereas pairs I and III have similar isolation dates and nosocomial transmission seems plausible, the two remaining pairs were isolated more than one year apart indicating that different transmission pathways might be involved.

**Table 2:** Description of four events of likely human-to-human transmission.

Case	Genetic distance (no. SNPs)	Lineage	Observations	Sampling date	Presence of IEC
I	0	L1	Concurrently hospitalized in the same ward	Mar 2015	−
				Mar 2015	−
II	1	L1	Both with respiratory infection, admitted to same section	Oct 2014	+
				Apr 2016	−
III	1	R	Same day in emergency unit	Sep 2014	−
				Oct 2014	−
IV	2	L3	Same home care	Jun 2015	−
				Aug 2016	+

Abbreviations: L1, Lineage 1; L2, Lineage 2; L3, Lineage 3; R, Remainder.

Comparison of the proportions of human and pig isolates clustering in the three most prevalent lineages of LA-MRSA CC398 in pigs (L1-L3), showed that human isolates were underrepresented in L2 (4% vs. 16%, *p* = 0.007), while no differences were observed in the two most prevalent lineages L1 and L3. The proportion of isolates clustering outside L1-L3 (i.e. the remainder) was higher (26% vs. 10%, *p* = 0.003) among human isolates than among isolates from pigs.

### Host-associated SNPs

None of the variable sites from the core genome was significantly associated with either human or pig origin after correction for multiple testing.

### Host association of predefined antimicrobial resistance and virulence genes

**Figure 3:**
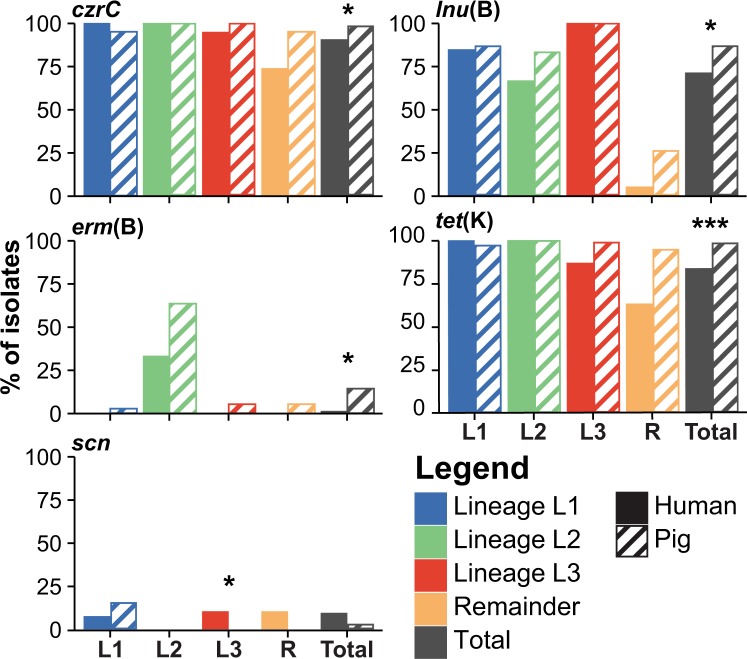
Presence of selected genes in LA-MRSA CC398 isolates from pigs and people with healthcare-associated infection. The paired bars show the prevalence of selected genes in isolates from humans (solid) and pigs (light) for each lineage (L1–L3), the remainder (R), and in total. The genes *czrC*, *erm*(B), *lnu*(B), and *tet*(K) confer resistance to cadmium/zinc, macrolide, lincosamide and tetracycline, respectively and IEC enables *S*. *aureus* to evade the human immune system. *Indicate significant *p* values as obtained from the analysis of predefined sets of genes and corrected for multiple testing. *P ≤ 0.05; **P ≤ 0.01; ***P ≤ 0.001.

The gene content of human and pig isolates was first compared using a set of predefined resistance and virulence genes. The genes were selected based on existing databases and evidence from previous studies on LA-MRSA CC398^11,18^. The results showed that human isolates had a significantly lower prevalence of the antimicrobial resistance genes *erm*(B), *lnu*(B), *tet*(K) and *czrC* conferring resistance to macrolides, lincosamides, tetracycline and cadmium/zinc, respectively (Table 3), after correction for multiple testing. Similar trends of lower prevalence of resistance genes in isolates from humans with significant associations prior to correction for multiple testing were also found in lineage L3 and the remainder (Fig. 3). In contrast, there was an opposite trend for a higher prevalence of the human variants of the IEC genes, *scn* and *sak*, in human isolates belonging to lineage L3, although the difference was non-significant after correction for multiple testing (Table 3). Moreover, IEC was found in one, four, and two isolates in L1, L3, and the remainder, respectively, including *scn* alone or in combination with other IEC genes, which indicates several independent acquisition events. In contrast, a unique IEC element containing *scn*, *chp*, *sak* and *sep* was found in a distinct cluster of six pig isolates within L1 suggesting that the element was acquired in a single event (Fig. 2).

**Table 3:** Genes with significant associations to human or pig origin.

Analysis/Gene	Analysed Group	No. (%) isolates		*p* value	
		Pig	Human	raw	corr.
**Predefined genes**					
*tet*(K)	All isolates	180 (98.4%)	61 (83.6%)	<0.001	<0.001
*erm*(B)	All isolates	25 (13.7%)	1 (1.4%)	0.0022	0.0262
*lnu*(B)	All isolates	159 (86.9%)	52 (71.2%)	0.0057	0.0438
*czrC*	All isolates	180 (98.4%)	66 (90.4%)	0.0067	0.0438
*vga*(A)V	All isolates	1 (0.6%)	4 (5.5%)	0.0243	n.s.
*sak*	L3	0 (0%)	4 (10.5%)	0.0058	n.s.
*scn* (IEC)	L3	0 (0%)	4 (10.5%)	0.0058	n.s.
*tet*(K)	L3	95 (99%)	33 (86.8%)	0.0071	n.s.
*erm*(C)	R	8 (42.1%)	1 (5.3%)	0.0188	n.s.
*dfrG*	R	19 (100%)	13 (68.4%)	0.0197	n.s.
*tet*(K)	R	18 (94.7%)	12 (63.2%)	0.0422	n.s.
**Accessory genes**					
SAPIG_RS05110^*‡^	All isolates	182 (99.5%)	59 (80.8%)	<0.001	<0.001
*tet*(K)	All isolates	180 (98.4%)	61 (83.6%)	<0.001	0.0063
*pre*	All isolates	180 (98.4%)	61 (83.6%)	<0.001	0.0063
*repN*	All isolates	180 (98.4%)	61 (83.6%)	<0.001	0.0063
SAPIG_RS15395^†‡^	All isolates	181 (98.9%)	63 (86.3%)	<0.001	0.0145
SAPIG_RS05110^*‡^	L3	96 (100.0%)	12 (31.6%)	<0.001	0.0197

Predefined genes with a significant raw *p* and accessory genes with significant *p* value after correction for multiple testing are shown. ^*^putative membrane protein; ^†^hypothetical protein; ^‡^genes could not be verified by read mapping. Abbreviations: corr., corrected for multiple testing; n.s., Not significant; L3, Lineage 3; R, Remainder.

Analysis of the presence/absence of the different genes in human isolates and their most closely related pig isolates showed that 100% (12/12), 24% (17/72), 52% (11/21) and 100% (7/7) of the human isolates lacking *erm*(B), *lnu*(B), *tet*(K) and *czrC*, respectively, were most closely related to a pig isolate that carried the corresponding gene, suggesting that they were lost during spread from the pig farms to the healthcare setting. In contrast, human isolates carrying these genes were always most closely related to a pig isolate also carrying the genes, suggesting that none of the human isolates have acquired the genes (Table 4). On the other hand, IEC (as represented by *scn*) was most likely acquired during or after transfer to the human host as the most closely related pig isolates of all seven positive human isolates were negative for the element.

**Table 4:** Gene gain and/or loss leading to human genotype.

Gene	Human/Pig genotype, N (%)				Most plausible scenario leading to human genotype	
	+/+	+/−	−/+	−/−	Presence through gain	Absence through loss
*tet*(K)	61 (84%)	0 (0%)	**12 (16%)**	0 (0%)	0/61 (0)	12/12 (100)
*erm*(B)	1 (1%)	0 (0%)	**17 (23%)**	55 (75%)	0/1 (0)	17/72 (24)
*lnu*(B)	52 (71%)	0 (0%)	**11 (15%)**	10 (14%)	0/52 (0)	11/21 (52)
*czrC*	66 (90%)	0 (0%)	**7 (10%)**	0 (0%)	0/66 (0)	7/7 (100)
*scn* (IEC)	0 (0%)	**7 (10%)**	0 (0%)	66 (90%)	7/7 (100)	0/66 (0)

The presence (+) or absence (−) of each gene in a human isolate (*n = *73) was compared to the presence or absence of the gene in the most closely related isolate from pigs (*n = *183). The proportion of human isolates which had acquired (Presence through gain) or lost (Absence through loss) the gene was calculated in all human isolates which displayed a different genotype than the most closely related pig isolate. The predominant scenario leading to the conclusion is highlighted in bold.

### Analysis of accessory genes

The pan-genome of all isolates as obtained from Prokka and Roary contained 4,019 genes. Of these, 4,008 could be confirmed by mapping of raw sequence reads to their reference sequences using Mykrobe predictor (0.27% false positives, 11/4,019). This re-mapping also led to an increase in the number of genes in the strict core-genome (genes present in all samples) from 2,029 to 2,302. Furthermore, 16.0% (642/4,019) of the genes were subsequently present in more samples than identified by Roary, while 3.6% (143/4,019) of the genes were found in fewer isolates.

After filtering the genes using a 1% frequency cut-off (i.e. present in more than two and less than 254 out of the 256 samples), 837 genes remained to be tested for association with either of the two host reservoirs. The results showed significant associations to pig isolates for five genes after correction for multiple testing, including the tetracycline resistance gene *tet*(K) together with two other genes (*pre* and *repN*) from the S0385 plasmid pS0385–1 (GenBank Accession Number NC_017334) containing *tet*(K) (Table 3). However, two genes (SAPIG_RS05110 and SAPIG_RS15395 on the S0385 chromosome [GenBank accession number NC_017333]) could not be confirmed by read mapping as they showed ambiguous mapping patterns with low numbers or lack of sequence reads that correlated with sequencing batches. This suggests that the significant results of these genes were due to a sequencing batch effect rather than traces of host adaptation. Lineage effects in GWAS can be circumvented by analysing associations in each lineage separately. This was specifically performed by analysing each of the three lineages L1-L3 and the remainder separately, with the result of only a single gene (SAPIG_RS05110, see above) in L3 being significantly associated with pigs after correction for multiple testing. This gene was also found associated with pigs in the analysis of all isolates (Table 3) but could not be confirmed by read mapping (see above).

### Phylogeny-based association analysis

The analysis of the accessory gene presence/absence data with lineage correction using treeWAS resulted in a significant terminal score for only one gene (SAPIG_RS05110) (Table 3), which corroborates the non-phylogeny-based analyses above. No other associations were found in any of the scores. The phylogeny-based analysis of core genome-wide SNPs using treeWAS showed no association of any SNP with either of the hosts.

*k*-mer-based analysis of raw sequencing reads using *pyseer* showed that *k*-mers with the lowest *p* values mapped to the proline-rich repetitive region of the *S. aureus* clumping factor B (*clfB)* gene in the ST398 reference genome S0385 (GenBank accession number AM990992), followed by several other genes including other so called microbial surface components recognizing adhesive matrix molecules (MSCRAMMs) with repetitive regions such as the three serine-aspartate repeat protein-encoding genes, *sdrC*, *sdrE*, and *sdrH* (Supplementary Fig. S1). However, validation by aligning assembled gene sequences as well as raw read mapping both produced inconclusive results likely due to low local sequence depth caused by a GC-bias and/or the repetitive nature of these regions. Alignment of the 96 high-coverage amplicons of this region showed no association of repeat size, phylogenetic clustering or specific variable sites to neither host, and thus the *k*-mer findings could not be confirmed.

## Discussion

This study shows that human LA-MRSA CC398 isolates from patients with healthcare-associated infections have their origin in pigs and that they are repeatedly introduced into healthcare facilities. The geographical distribution of healthcare-associated infections was similar to the distribution of LA-MRSA CC398 in the pig population, supporting that introduction of LA-MRSA CC398 into the healthcare system is due to spillover from nearby pig farms via human-to-human^13,23^ or environmental transmission chains^24–26^. This is in line with an earlier Danish study, which showed that most LA-MRSA CC398 isolates from Danish infections were more closely related to Danish pig isolates than to isolates of international origin^2^, and with a Dutch study where people without livestock contact were shown to carry variants known from the local livestock population^12^.

We found similar proportions of L1 and L3 isolates in pig farms and people with healthcare-associated infections, suggesting that they have the same capacity for spread into healthcare facilities. However, isolates from human infections were underrepresented in L2 and overrepresented among the remainder. An overrepresentation of particular lineages in LA-MRSA CC398 isolates from humans has been observed before^15,27^ and this can be indicative for lineages better adapted to colonize and infect humans. Indeed, the remainder are known to have a lower prevalence of antimicrobial resistance genes than the three predominant lineages in Danish pigs^11^. The lower fitness costs due to absence of these resistance genes may provide an advantage in the human environment in absence of the selection pressure exerted by the corresponding antimicrobial compounds. However, L2 does not have more resistance genes than L1 and L3^11^, but still seems to cause fewer infections in humans, indicating that other factors may play a role in determination of the frequency of human infection.

Comparison of LA-MRSA CC398 genomes from pigs and humans revealed a lower prevalence of predefined antimicrobial resistance genes in human isolates. All of these genes are encoded on mobile genetic elements (MGEs) including plasmids for *erm*(B), *lnu*(B) and *tet*(K)^28–30^ and the highly dynamic staphylococcal cassette chromosome *mec* (SCC*mec*) element for *czrC* and in most cases also *tet*(K)^31^. The lower prevalence of these genes in humans could be due to transmission of isolates that already lack these genes or to gain and loss of MGEs during transmission from pig farms to the human environment. Our analysis supports the latter scenario, with frequent loss of antimicrobial resistance genes and gain of IEC in human isolates. The lost genes confer resistance to antimicrobials commonly used in the pig production. Thus, the genes are likely lost because they do not provide a fitness advantage in absence of the corresponding antimicrobial compounds in the human hosts. Likewise, LA-MRSA CC398 is generally considered as poorly adapted for transmission among humans, in particular because of the absence of the ΦSa3 prophage carrying IEC^18,32^. The *scn* gene is always present in IEC^33^ and encodes the staphylococcal chemotaxis inhibitor protein (SCIN), which plays an important role in the bacterium’s ability to evade the innate immune response in humans^34^. Our analysis indicates that IEC and *scn* have been acquired by several human strains. Taken together, these results show that LA-MRSA CC398 is capable of repeated re-adaptation to the human environment by acquisition and loss of genes carried on MGEs.

In order to confirm the absence of genomic changes other than those related to MGEs, a highly sensitive *k*-mer-based GWAS was performed. This analysis showed highly significant associations of genomic regions with the hosts (Supplementary Fig. S1), but these associations could not be confirmed in the subsequent analysis of sequenced amplicons of these regions. This might be due to the regions showing sequence patterns known to cause sequencing biases such as palindromes, inverted repeats, long homopolymers or other repetitive regions^35–37^, highlighting the importance of uniform sequencing data and the need to confirm positive results when using such highly sensitive bioinformatic methods as *k*-mer-based GWAS from short read sequencing data.

Four pairs of closely related human isolates were identified in the phylogenetic analysis and subsequent epidemiological investigations showed that in all cases, nosocomial transmissions may have occurred, although there is differences in the gene content of some of the isolates and the large timespan between the isolation dates in two of the four pairs may suggest different transmission pathways. These findings are consistent with a previously reported outbreak of LA-MRSA CC398 in an intensive care unit^10^, and together they provide strong evidence of human-to-human transmission of LA-MRSA CC398 within the Danish healthcare system. However, whether the transmissions were directly between humans or via surfaces or items remains unclear, and the phylogenetic analysis showed that three of the four pairs occurred in different pig-associated lineages indicating that nosocomial transmission of LA-MRSA CC398 is rare and sporadic.

In accordance with the here substantiated scenario of a repeated spillover of random LA-MRSA CC398 isolates from livestock to the community, the number of CO infections from 2007 to 2016 increased in parallel with the prevalence in Danish pig farms. On the other hand, the numbers of HO and HACO infections remained low in the same period, indicating that LA-MRSA CC398 is successfully kept out of healthcare institutions thanks to effective measures such as the Danish screening program during admission to healthcare institutions and a high standard of infection control measures in general.

The present study is limited by the relatively low number of isolates from healthcare-related infections, which represent only a small proportion of those caused by LA-MRSA CC398 in the community. However, the collection represents an unbiased nationwide sample of isolates from cases where strong epidemiological data ascertains the absence of a link to livestock. Furthermore, the focus on isolates from human infections might lead to an underestimation of nosocomial transmission since transmission from and to asymptomatic carriers, including e.g. healthcare workers who might have indirect pig contact, is not detected.

In conclusion, the present study showed that the vast majority of healthcare-associated infections with LA-MRSA CC398 in humans can be explained by repeated spillover of random isolates from pig farms. This suggests that a reduction of LA-MRSA CC398 in pigs would be mirrored in a reduction of infections in high-risk patients in hospitals and other healthcare units. Furthermore, the present study identified at least two healthcare-associated transmission events and showed that the host transition of LA-MRSA CC398 from pigs back to humans was accompanied by repeated, independent loss of antimicrobial resistance genes and occasional gain of the ΦSa3 prophage carrying IEC genes. Over time and with increased occurrence, this process of parallel evolution could lead to re-adaptation of LA-MRSA CC398 to the human host and eventually the emergence and spread of lineages more adapted (and potentially more virulent) to humans. A continued surveillance of LA-MRSA CC398 in both pigs and humans is therefore essential for early detection of and action against such potentially emerging lineages.

## Methods

### Isolate collection and classification

All primary LA-MRSA CC398 isolates recovered from Danish patients who had a healthcare-associated (i.e. HO or HACO) infection during a 3-year period from January 2014 through December 2016 were included in this study and subjected to whole-genome sequencing as described below. The LA-MRSA CC398 isolates were selected based on the national MRSA registry and strain repository at Statens Serum Institut in Copenhagen. All data collection and analyses were in accordance with guidelines and regulations as approved by the Danish Data Protection Agency (protocol number 2001-14-0021). The same protocol approves for use of Danish surveillance data, hence obtaining informed consent from all subjects was not necessary. All isolates were typed at Statens Serum Institut as part of the national MRSA surveillance program^4^. For each patient, the infection was defined as LO, CO, HO, or HACO through review of the medical records, using the criteria described above. To describe temporal trends of LA-MRSA CC398 in Denmark, all primary LA-MRSA CC398 isolates recovered from human infections from 01 January 2007 through 31 December 2016 were used.

### Whole-genome sequencing and analyses

A schematic overview over the methods used in this study can be found in Supplementary Fig. S2. The human LA-MRSA CC398 isolates identified in this study were whole-genome sequenced on an Illumina MiSeq (*n = *27) with 2 × 251 bp paired-end reads or Illumina NextSeq. 550 (*n = *46) with 2 × 151 bp paired-end reads after library preparation using the Nextera XT DNA Library Preparation Kit (Illumina, San Diego, USA). The sequences were compared to 183 LA-MRSA CC398 genomes from pigs representing all available LA-MRSA CC398 isolates from a nationwide survey in Danish breeding farms (46 isolates) and production farms (137 isolates) in 2014^11^. Single nucleotide polymorphisms (SNPs) were called and filtered using NASP version 1.0^38^ and a maximum-likelihood phylogenetic tree was calculated in IQ-TREE version 1.6.1^39^ using default settings with a GTR substitution model after removal of recombinant regions, as described previously^11^. The tree was rooted according to Sieber *et al*.^11^ and the three predominant lineages of LA-MRSA CC398 in Danish pigs were determined according to Sieber *et al*.^11^. The genetic distance between isolates was calculated as the number of sites that differ between each pair of sequences in the detected core genome.

To determine whether a genotype in human isolates was the result of transmission of a pig isolate with the same genotype or whether the genotype evolved during this transmission, the most probable scenario for the human genotype was inferred by comparison of each human isolate to its most closely related pig isolate.

### Genome-wide association analyses

Gene presence was determined using Mykrobe predictor version 0.4.3^40^ with reference graphs generated either from a predefined set of genes containing all entries from the ResFinder^41^ and VirulenceFinder^42^ databases (http://www.cge.dtu.dk, accessed 01 October 2018) and the *czrC* gene encoding resistance to cadmium and zinc (GenBank accession no. KF593809), or from the pan-genome of all isolates as determined by Prokka^43^ and Roary^44^ using SPAdes-assembled^45^ genomes and default settings. Genes classified as present by Mykrobe predictor were further filtered for median depth (≥5×).

A total of 3,245 high quality SNPs were identified in the core-genome using NASP (see above) and analysed for association with either of the host groups using Fisher’s exact tests.

Phylogenetic tree-based genome-wide association analyses were performed in the R-package treeWAS^46^ for the presence/absence of genes and SNPs and using the python software *pyseer*^47^ for a *k*-mer-based analysis to account for genomic differences other than presence/absence of detected genes or SNPs. The latter was performed on *k*-mers produced from raw sequencing reads with a minimum per *k*-mer count of eight per sample using a linear mixed effects model with a similarity matrix inferred from the maximum-likelihood phylogenetic tree. The *k* was chosen as the median of the range of *k*s which the *k*-mer generator distributed with *pyseer* uses by default (*k = *54). Significant *k*-mer hits were annotated by mapping to the *S. aureus* CC398 reference chromosome S0385^48^.

### PCR and sequencing of amplicons

The *clfB* region with the lowest detected *p* value was chosen as a representative to be investigated by targeted sequencing of PCR amplicons in 96 randomly sampled isolates. The proline-rich region of the *clfB* gene was amplified using KAPA HiFi HotStart ReadyMixPCR Kit in 25 μL PCR reactions (Kapa Biosystems, Wilmington, USA) with forward (5′-CCCAAATGACTCTAACCT-3′) and reverse (5′-GCTCTTATCTCCTGTTTCT-3′) primers designed from the *S. aureus* reference chromosome S0385^48^ using CLC Genomic Workbench Version 12.0 (QIAGEN, Aarhus, Denmark), and the following PCR conditions: 95 °C for 3 min, 25 cycles of 95 °C for 30 sec, 58 °C for 30 sec, 72 °C for 2 min, and final extension at 72 °C for 7 min. The obtained amplicons were depleted of excess primer using Beckman Coulter AMPure XP beads and libraries were prepared with the Nextera XT DNA Library Preparation Kit (Illumina, San Diego, USA). After normalization and pooling, libraries were sequenced on an Illumina MiSeq instrument with 2 × 251 bp paired-end reads. The resulting sequences were assembled using SPAdes^45^ with “careful” option and contigs were filtered for a minimum average depth of 500× before a consensus sequence for each isolate was generated in Geneious version 11.1.5 (Biomatters Ltd., Auckland, New Zealand). The resulting sequences of the *clfB* region were aligned using MAFFT version 1.3.7^49^ as implemented in Geneious (Biomatters Ltd.) to identify length variations and variable sites in the proline-rich repeat region.

### Statistical analyses

Statistical analyses were performed in R version 3.5.0^50^. *p* values were obtained from Fisher’s exact tests and corrected for multiple testing using false discovery rate (FDR) where appropriate.

## Supplementary information

Supplementary Information

## Data Availability

The whole-genome sequence data generated in this study have been submitted to the European Nucleotide Archive under BioProject accession number PRJEB25608. Datasets analysed and scripts used during the current study are available in the Sourceforge repository, https://sourceforge.net/projects/mrsa-cc398-haco-vs-pigs/files.
