# Supplementary material for: Genome investigations show host adaptation and transmission of LA-MRSA CC398 from pigs into Danish healthcare institutions
Source: Sci Rep. 2019 Dec 9;9:18655. doi: 10.1038/s41598-019-55086-x (PMC6901509; doi:10.1038/s41598-019-55086-x)
Supplement: Supplementary file 1 — Supplementary Information [file 41598_2019_55086_MOESM1_ESM.pdf]

Supplementary information to:

Genome investigations show host adaptation and transmission of LA-MRSA CC398 from pigs into Danish healthcare institutions

Raphael Niklaus Sieber[1]*, Anders Rhod Larsen[1], Tinna Ravnholt Urth[2], Søren Iversen[1], Camilla Holten Møller[2], Robert Leo Skov[1], Jesper Larsen[1], Marc Stegger[1]*

[1] Statens Serum Institut, Department of Bacteria, Parasites & Fungi, Artillerivej 5, 2300 Copenhagen S, Denmark

[2] Statens Serum Institut, Department of Infectious Disease Epidemiology & Prevention, Artillerivej 5, 2300 Copenhagen S, Denmark

*Address correspondence to Raphael N. Sieber, rasi@ssi.dk, or Marc Stegger, mtg@ssi.dk.

Supplementary Figure S1

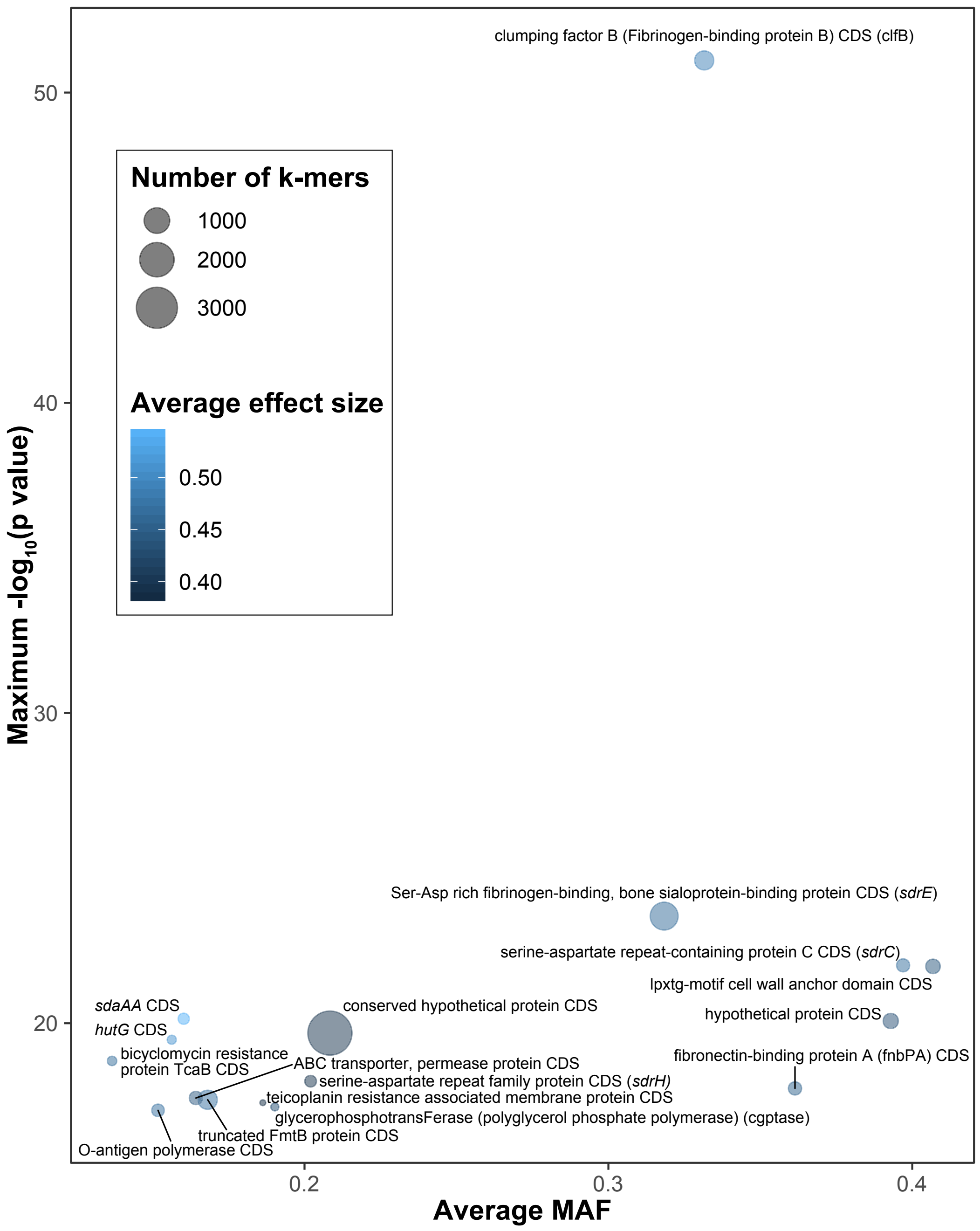

Supplementary Figure S1: Genomic regions with human host associations. The figure shows *k*-mer hits as obtained in *pyseer* and their mapping to sequence annotations in the ST398 S0385 reference genome. Only hits with a *p* value $\leq 10^{-17}$ are shown. Gene hits are shown as circles placed according to the lowest p value of any *k*-mer with a hit in a gene (y-axis) and the average minor allele frequency (MAF) indicating the commonness of the hits (x-axis). The circles are sized according to the number of *k*-mer hits mapped to the region and colored according to the effect size indicating the distribution among the phenotypes.

Supplementary Figure S2

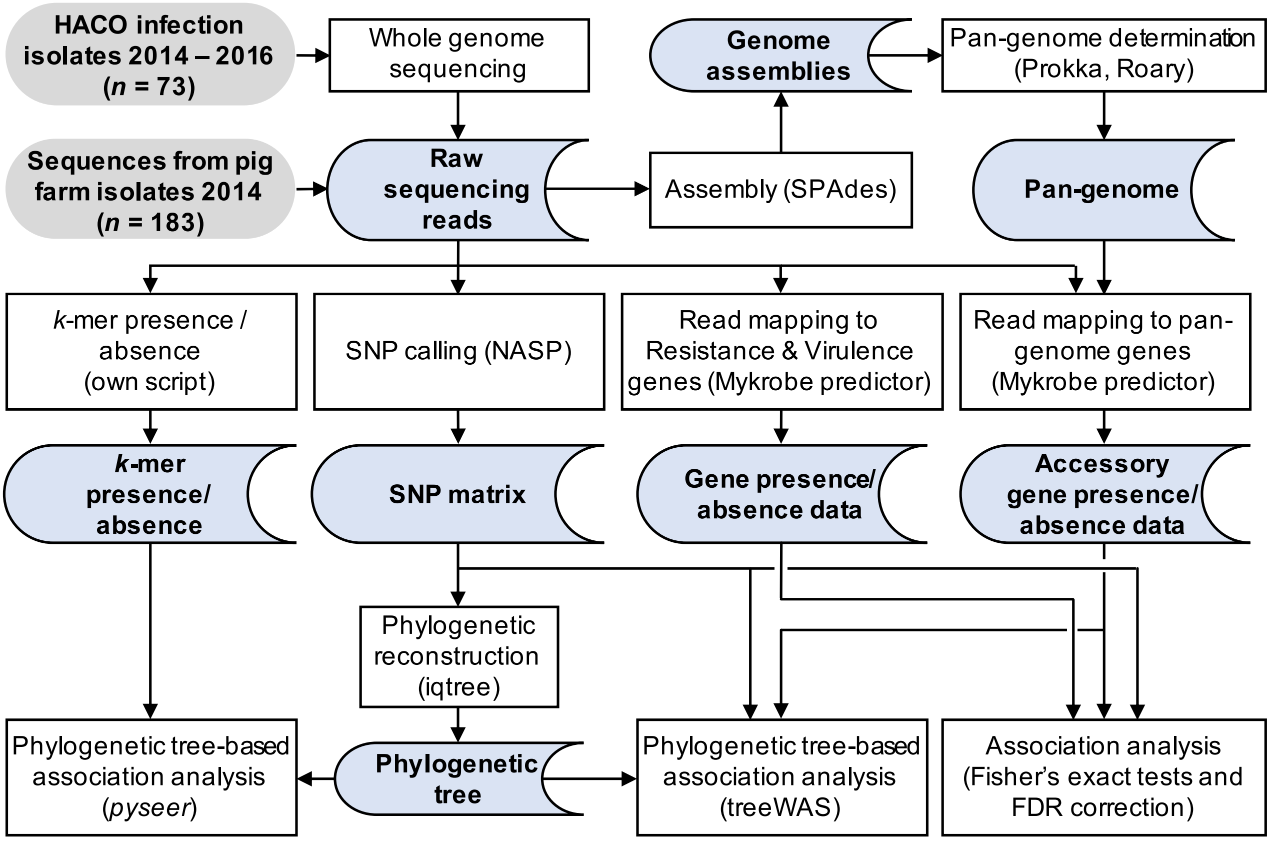

Supplementary Figure S2: Study overview. Initial datasets (grey boxes), analyses (white boxes) and intermediate datasets/results (blue boxes) are connected according to their use in the study.
